# Beefy red asymptomatic penile lesion: unusual presentation of squamous cell carcinoma^☆^^☆☆^

**DOI:** 10.1016/j.abd.2020.06.019

**Published:** 2021-03-15

**Authors:** Seema Rani, Kabir Sardana, Arvind Ahuja

**Affiliations:** Dr Ram Manohar Lohia Hospital, New Delhi, India

Dear Editor,

A 65-year-old male was presented with a lobulated beefy red plaque on his penile shaft with a two-month duration. During the physical examination there was a well-defined non-tender erythematous, lobulated, sessile with a growth of size of approximately 5 × 3 cm involving glans, coronal sulcus and the penis shaft (Fig. 1). There was no lymphadenopathy. Initially, the patient had mild pruritus and with subsequent interval of time noticed tiny penile growth which gradually progressed to an increased present size in a two-month duration, along with some difficulty to retract the prepuce. No history of dysuria was present. He was otherwise in good health. His family, medical and social history was non-contributory. He had history of smoking for 20 to 25 years, and quit smoking for the last 5-year period.

**Figure 1:**
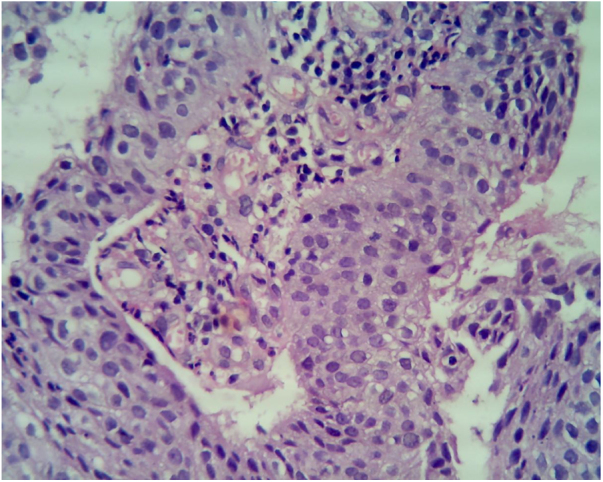
Well defined, erythematous, fleshy, lobulated, sessile growth involving glans, coronal sulcus and shaft of penis.

Even though the patient denied any sexual contact, a differential diagnosis of condyloma acuminate, condyloma lata and granuloma inguinale were kept with other remote differentials, which were verruciform xanthoma and squamous cell carcinoma. All biochemical and haematological investigations including lipid profile, liver function test, kidney function test, serum electrolytes, routine and a microscopic examination of urine and complete blood count were within normal limit. Dark ground microscopy for *Treponema pallidum* and tissue-smear for Donovan bodies were negative. A serological test for syphilis and Elisa for HIV Type 1 and 2 were negative. The initial biopsy specimen reported condyloma accuminata with squamous cell showing loss of polarity without any cytological atypia. Repeat biopsy was sent, which was consistent with squamous cell carcinoma with papillary features (Fig. 2). An ultrasound of the abdomen and pelvis was done, which were normal. A surgical excision was done and this re-confirmed the diagnosis of squamous cell carcinoma in situ.

**Figure 2:**
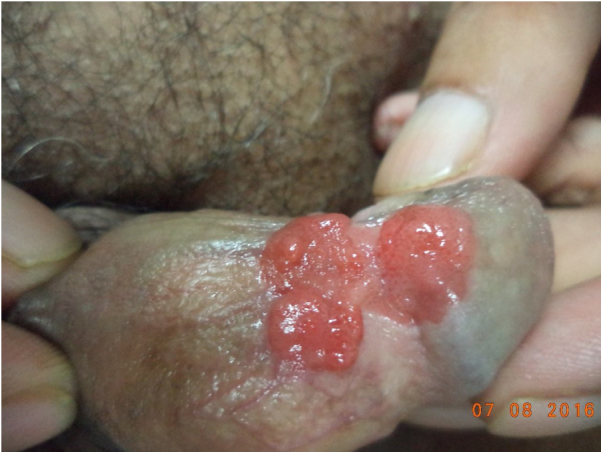
Beefy red asymptomatic penile lesion: unusual presentation of squamous cell carcinoma. Atypical cells with nuclear overcrowding, loss of polarity, lack of surface maturation, high N:C ratio, hyperchromatic nuclei, and multiple mitotic figures. Focal clear cell change is noted at the junction of atypical epithelium and normal epithelium (Hematoxylin & eosin, ×40).

The asymptomatic beefy red lobulated plaque on the penile region has various differentials. As the patient had a negative tissue smear, the possibility of Condyloma acuminata and verruciform xanthoma was kept. Verruciform xanthoma is a rare entity that principally involves the mouth. Genitalia are the next most frequently affected area, where it presents itself as a painless, yellow-brown, or red, verrucous, sessile, or papillary plaque. As the patient had no regional lymph node enlarged, squamous cell carcinoma was not considered as a first differential. Also, there was no pre-existing lesion which can predispose to the development of squamous cell carcinoma. Nonetheless, the biopsy confirmed a diagnosis of squamous cell carcinoma in situ. Squamous cell carcinoma is the most common tumor of the penis, and presents itself either as a flat growth, infiltrating, or papillary growth, or a hard painless lump. Penile carcinoma is mainly a localized disease (39%), with carcinoma-in-situ making up 37% of the total penile carcinoma cases.^1^, ^2^ It is most commonly diagnosed in elderly patients (50–70 years).^2^ It has a higher incidence in the developing countries and is most commonly on the glans penis (48%), followed by the prepuce (25%), the glans and the prepuce (9%), coronal sulcus (6%) and on the shaft (< 2%). Ninety-five percent of these malignancies are squamous cell carcinomas.^3^

Our case was initially seen in the STD referral center, hence the diagnosis of squamous cell carcinomas was not considered as a first differential. The lack of any other predisposing factors and lack of regional lymph node involvement might have been a reason for a clinical diagnosis of condyloma acuminate and granuloma inguinale. The short history with no associated risk factors for malignancy is another reason for not considering squamous cell carcinoma.

The treatment of squamous cell carcinomas in situ is local excision, though partial penectomy and monthly follow-ups for at least 1 year is appropriate for patients with small, well-differentiated primary tumors. Patients who have large or moderately to poorly differentiated primary tumors probably should undergo partial or total penectomy and immediate ilioinguinal lymphadenectomy.^4^ In the absence of inguinal metastases, patients with invasive squamous cell carcinomas of the penis involving the glans or the distal part of the shaft who undergo adequate partial amputation have a long-term survival rate of 70%–80%. Of patients with involved lymph nodes, 40%–50% can be cured with lymph node dissection, whereas untreated patients usually die within 2–3 years.^5^ In this case, partial penectomy was done.

This case elegantly demonstrates the possibility of squamous cell carcinoma even without any predisposing factors, which can mimic common sexually transmitted disease, herein Condyloma acuminata and granuloma inguinale. Needless to say, in doubtful cases a repeat biopsy is warranted, as demonstrated in our case, to enable correct management in such circumstances.

## Financial support

None declared.

## Authors' contributions

Seema Rani: Approval of the final version of the manuscript; collection, analysis, and interpretation of data; critical review of the literature.

Kabir Sardana: Approval of the final version of the manuscript.

Arvind Ahuja: Approval of the final version of the manuscript; collection, analysis, and interpretation of data.

## Conflicts of interest

None declared.
